# Fluconazole Resistance among Oral *Candida* Isolates from People Living with HIV/AIDS in a Nigerian Tertiary Hospital

**DOI:** 10.3390/jof3040069

**Published:** 2017-12-08

**Authors:** Iriagbonse I. Osaigbovo, Patrick V. Lofor, Rita O. Oladele

**Affiliations:** 1 Department of Medical Microbiology, School of Medicine, University of Benin, Benin City PMB 1154, Nigeria; loforpa@yahoo.com; 2 Department of Medical Microbiology, University of Benin Teaching Hospital, Benin City PMB 1111, Nigeria; 3 Division of Infection, Immunity and Respiratory Medicine, Faculty of Biology, Medicine and Health, The University of Manchester, Manchester M139PL, UK; rita.oladele@postgrad.manchester.ac.uk

**Keywords:** HIV, *Candida* species, fluconazole resistance, oropharyngeal candidiasis, Nigeria

## Abstract

Oropharyngeal candidiasis, a common fungal infection in people living with HIV/AIDS (PLWHA), arises from *Candida* species colonizing the oral cavity. Fluconazole is the preferred treatment and is often used empirically. Few studies have investigated the prevalence of fluconazole resistance in Nigeria. This study aimed at determining the burden of fluconazole resistance among *Candida* species in the oral cavities of PLWHA. We sampled the oral cavities of 350 HIV-infected adults and an equal number of HIV-negative controls. *Candida* isolates were identified using germ tube tests, CHROMagar *Candida* (CHROMagar, Paris, France), and API *Candida* yeast identification system (BioMérieux, Marcy-l’Étoile, France). Fluconazole susceptibility was determined using the Clinical and Laboratory Standards Institute disc diffusion method. Data were analysed using SPSS version 21 (IBM, New York, NY, USA). The significance level was set at *p* ≤ 0.05. The isolation rates for *Candida* amongst HIV-infected subjects and controls were 20.6% and 3.4%, respectively (*p* < 0.001). In PLWHA, *Candida albicans* was most frequently isolated (81.3%) and fluconazole resistance was present in 18 (24%) of the 75 *Candida* isolates. Resistance to fluconazole was present in half of the non-*albicans Candida* isolates. Fluconazole resistance is prevalent among oral *Candida* isolates in PLWHA in the study area with a significantly higher rate among non-*albicans Candida* spp.

## 1. Introduction

Oropharyngeal candidiasis (OPC) is the most prevalent opportunistic fungal infection in people living with HIV/AIDS (PLWHA) [1]. The causative agent, *Candida* spp., is part of the oral mycobiome. Carriage rates vary with age and immune status, being higher in the immunocompromised host. Although not a life-threatening condition in itself, OPC significantly lowers the quality of life of PLWHA. It manifests with local discomfort and altered taste sensation. Severe cases can interfere with administration of medication and nutritional intake. OPC may be complicated by *Candida* oesophagitis which presents with dysphagia and retrosternal pain. Clinical variants of OPC include the pseudomembranous, erythematous, and hypertrophic types, as well as *Candida*-associated lesions, such as angular cheilitis, denture stomatitis, and median rhomboid glossitis [2]. OPC requires distinction from other oral mucous pathologies which may present in a similar fashion, including chemical and thermal burns, traumatic ulceration, mucous patches of syphilis, drug reactions, erosive lichen planus, pernicious anaemia, and burning mouth syndrome [3].

Globally, 50% of HIV-infected individuals and 90% of those with AIDS suffer from OPC [4]. With approximately 4 million cases of HIV per year, this equates to about 2 million cases of OPC annually [4].

*Candida albicans* is the most frequently implicated pathogen. However, non-*albicans Candida* (NAC) species have been increasingly isolated from HIV-infected patients with OPC and this has been attributed to repeated exposure to antifungal agents [5,6,7]. The significance of non-*albicans* species lies in their frequent association with drug resistance as well as recurrent and refractory infections [8]. Fluconazole, often used empirically, is the drug of choice for treatment of OPC in PLWHA [8,9]. Widespread use has resulted in the emergence of azole resistance in previously susceptible strains of *C. albicans* and in the selection of *Candida* species intrinsically less susceptible to fluconazole [10]. This could hamper the empirical use of fluconazole as a treatment for OPC.

With a population estimate of over 170 million and an adult HIV prevalence of 3.4%, Nigeria has the second heaviest burden of PLWHA in Africa; invariably OPC is frequently encountered [11]. Fluconazole resistance, which could constitute a problem in its management, is underreported in Nigeria. In this study, we investigated the burden of fluconazole resistance in the study location.

## 2. Materials and Methods

We carried out a descriptive, cross-sectional study in the President’s Emergency Plan for AIDS Relief (PEPFAR) clinic in the University of Benin Teaching Hospital (UBTH). UBTH is an 850-bed tertiary hospital located in Benin City, the capital of Edo State, Nigeria. It serves as a referral centre for Edo state and neighbouring regions.

The study was conducted in accordance with the Declaration of Helsinki, and the protocol was approved by the institutional Ethics Committee (ADME/E 22/A/VOL. VII/906). Informed written consent was obtained from each patient after adequate explanation of the study and its objectives.

A systematic random sampling technique was employed to select consenting participants amongst HIV-infected adults attending the clinic. Non-consenting persons, diabetics, smokers, and persons with dentures were excluded from the study. A total of 350 subjects were recruited. An equal number of control subjects (HIV negative) were randomly selected from voluntary blood donors in the same facility.

The buccal mucosa, tongue, palate, and oropharynx (including any OPC lesions present) of each participant were swabbed with sterile cotton-tipped swab sticks. Swab specimens were inoculated without delay onto Sabouraud dextrose agar (Oxoid, Hampshire, UK) containing chloramphenicol (0.05 g/L) and CHROMagar *Candida* (Chromagar, Paris, France). They were incubated at 37 °C for 48 h. Colony morphology and colour on CHROMagar *Candida* were noted.

The performance of each batch of CHROMagar *Candida* was tested using quality control strains *C. albicans* American Type Culture Collection (ATCC) 90028 and *C. parapsilosis* ATCC 22019.

Typical yeast colonies were Gram stained and examined microscopically for confirmation. All isolates were subjected to a germ tube test. Single colonies from subculture were suspended in human serum and incubated for 90 min at 37 °C. Wet mounts were prepared from the suspension and examined under a microscope for germ tube formation. Isolates which were germ tube-positive were presumed to be *Candida albicans*.

The colour of the various colonies on CHROMagar *Candida* were noted and used in the presumptive identification of isolates. *Candida dubliniensis*, which also gives a positive germ tube test, was excluded and differentiated from *C. albicans* using colour on CHROMagar as described by Messeir et al. [12].

The final identities of isolates were subject to confirmation using API *Candida* yeast identification system (BioMérieux, Marcy-l’Etoile, France) in accordance with manufacturer’s instructions.

Antifungal susceptibility to fluconazole was performed on identified isolates as outlined in the CLSI M44-A2 document for disc diffusion susceptibility testing in yeasts using Mueller Hinton + 2% glucose + 0.5 µg/mL methylene blue dye (GMB) agar and 25 µg fluconazole-impregnated discs (Oxoid, Hampshire, UK) [13]. The zones of inhibition were measured and the results interpreted according to CLSI interpretive criteria as follows: sensitive ≥ 19 mm; susceptible dose-dependent (S-DD) = 15–18 mm; resistant ≤ 14 mm.

OPC was taken to be the presence of adherent white plaques on oral mucosal surfaces and the growth of yeasts on culture obtained from the oral cavity. Colonisation referred to the growth of yeasts on culture in the absence of oral lesions.

The clinical and laboratory data were analysed using the SPSS version 21 computer software (IBM, New York, NY, USA). Frequency tables were used to present data. Chi square test or Fisher’s exact test was used to test the association between categorical variables, as applicable. *p*-values < 0.05 were considered statistically significant.

## 3. Results

### 3.1. Participants’ Characteristics

A total of 350 adults comprising 262 females (74.9%) and 88 males (25.1%) with HIV infection participated in the study. Participants ranged in age from 18 to 75 years (Mean 41.6 ± 10.0 years). The mean duration of time from diagnosis of HIV infection was 5.1 ± 3.5 years with a range of 0–20 years.

The CD4 counts of study participants ranged from four to 1523 cells per microliter. The mean cell count was 442.7 ± 285 cells/µL and the median was 401.5 cells/µL. A CD4 cell count below 200 cells/µL was found in 73 participants (20.9%).

Three hundred and twenty six subjects were on highly active antiretroviral therapy (93.1%). Forty seven participants had previous exposure to fluconazole therapy (13.4%). None of them was on prophylactic antifungal agents at the time of recruitment and none had used fluconazole in the preceding six weeks. Fifty-three participants had a past history of OPC (15.1%). There was no evidence of concurrent or past systemic mycoses in any of the participants.

Seventeen study subjects’ (4.9%) lesions were consistent with a clinical diagnosis of OPC.

### 3.2. Species Distribution and Fluconazole Susceptibility of Isolates

Oral swabs from 72 participants were positive on culture giving an isolation rate for *Candida* species of 20.6%. Of these, 55 were colonised, but asymptomatic (76.4%), while 17 had OPC (23.6%). *Candida* species were isolated from all the patients with clinical lesions.

Seventy five *Candida* isolates were recovered. The most frequently isolated species was *Candida albicans*, numbering 61 (81.3%). Among the NAC, *C. parapsilosis* was the most common, five (6.7%); followed by *C. glabrata*, four (5.3%); *C. tropicalis*, two (2.7%); *C. krusei*, two (2.7%) and *C. famata*, one (1.3%). Three participants had mixed growth of *Candida* (4.2%); each had *C. albicans* in addition to a NAC species. *Saccharomyces cerevisiae* plus *C. albicans* were isolated from one participant (1.4%).

Of the 75 isolates from the HIV infected participants, 18 were resistant to fluconazole (24%), four were susceptible-dose dependent (5.3%), and 53 were susceptible (70.7%). All isolates from the control group were susceptible to fluconazole (100%). Table 1 shows the fluconazole sensitivity pattern of different species of *Candida* isolated from PLWHA.

### 3.3. Fluconazole Resistance and Associated Factors

The factors found to be significantly associated with fluconazole resistant isolates are highlighted in Table 2.

**Figure 1:**
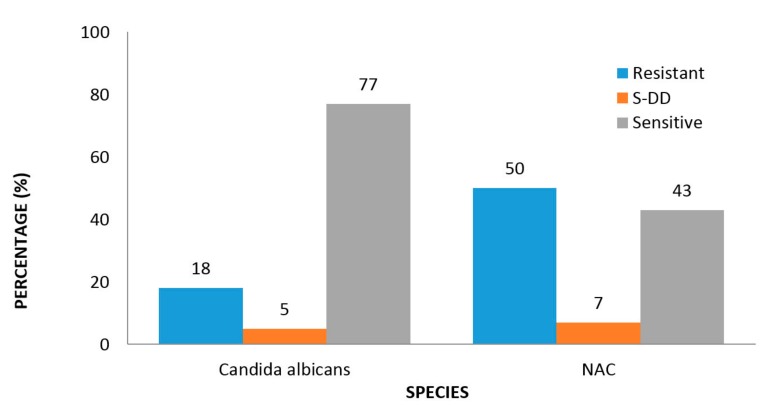
Fluconazole sensitivity of *Candida* isolates in PLWHA.

As shown in Figure 1, 18.0% of all *C. albicans* isolates from PLWHA were resistant to fluconazole compared with 50.0% among the non-*albicans Candida* species and this difference was statistically significant (*p* = 0.032, Table 2).

**Figure 2:**
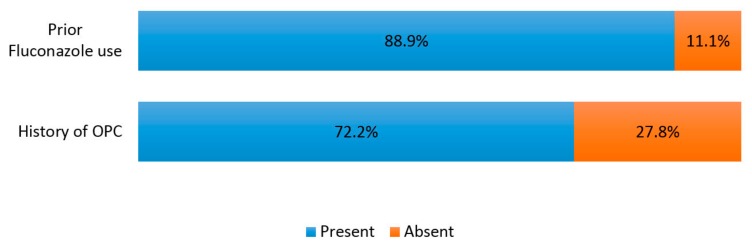
Prevalence of prior fluconazole use and history of OPC in fluconazole-resistant isolates.

Of the 18 fluconazole resistant isolates, 16 (88.9%) were from individuals with prior exposure to the drug. Figure 2 highlights the prevalence of prior exposure to fluconazole and history of OPC among fluconazole resistant isolates.

Seventeen (4.9%) of the PLWHA had OPC and *Candida* species were isolated from all. Of these isolates, 16 (94.1%) were *C. albicans* and one was *C. tropicalis* (5.9%). Three (17.6%) of the isolates were fluconazole resistant—all *C. albicans*—and from patients with a history of previous fluconazole use.

### 3.4. Control Subjects

Three hundred and fifty apparently healthy HIV-negative voluntary blood donors were sampled as control subjects and 12 (3.4%) were colonised orally with *Candida* spp. Isolation of *Candida* was significantly higher among PLWHA when compared to the HIV-negative controls (χ^2^ = 48.701, N = 700, *df* = 1, *p* < 0.001).

None of the control subjects had OPC. All 12 isolates from the control subjects were *Candida albicans* (100%): all were sensitive to fluconazole.

## 4. Discussion

Few studies have elucidated the epidemiology of OPC in Nigeria; fewer still have documented the fluconazole sensitivity pattern of oral isolates of *Candida* species [14,15,16,17]. This work contributes to the important, yet neglected, discourse on mycoses and their treatment, especially in immunocompromised populations in an African setting.

We observed resistance to fluconazole in 24.0% of oral *Candida* isolates from PLWHA (17.6% among isolates from symptomatic patients) in Benin City, Nigeria; 50% of NAC were resistant to the antifungal agent.

The prevalence in this study is higher than rates obtained elsewhere in Nigeria [16,17]. An important observation was that previous studies lacked information about possible contributory factors, such as past fluconazole use and previous OPC among subjects. In our study, 88.9% of resistant isolates were obtained from PLWHA who had prior exposure to fluconazole and 72.8% had a history of OPC. These variables were significantly associated with fluconazole resistance. Therefore, we can assume the differences in study populations with respect to these variables account for the discordance in rates. Other possible explanations for the difference in rates include methodological, temporal, and regional variations.

The disc diffusion protocol, as outlined in CLSI M44-A2, was employed in this study. We chose this approach because it is cheap, quick, and reproducible; moreover, it correlates well with results obtained by the reference broth microdilution method [18,19]. Conversely, other studies employed a broth macrodilution method. Enwuru et al. [20], however, demonstrated good correlation between broth macrodilution and disc diffusion.

A cursory appraisal suggests a temporal rise in fluconazole resistance: 9.5% in Lagos (2008), 11.7% in Abakiliki (2011), and 24% in Benin (2017) [16,17]. However, repeated surveillance in the respective study locations would be required to validate such a claim.

The observed disparity in rates could also be accounted for by regional variation. Fluconazole resistance varies widely within and between regions. Recent studies conducted in other African countries show that resistance to fluconazole in oral *Candida* isolates ranges from 5.0% to 50.0% [7,21,22,23]. In Ethiopia, Wabe et al. [21] observed a resistance rate of 11.9%, although only *C. albicans* isolates were considered in that study. Including NAC species might have raised this figure since higher rates of resistance commonly occur among NAC species when compared to *C. albicans* [8]. Abrantes et al. did not observe this difference among isolates from Cameroon and South Africa, but it was a significant finding in our work [23]. Current data from outside the continent estimate resistance to fluconazole among oral *Candida* isolates at 0% in Turkey, 3.4% in Texas, USA, 2.9% in Taiwan, 5.6% in China, and 39.1% in India [5,24,25,26,27]. The variability within and between regions highlights the need for routine local surveillance.

Fluconazole is commonly used for prophylaxis and treatment of both mucocutaneous and invasive fungal infections in many parts of the world [28]. This is due to its favourable pharmacokinetic profile, low toxicity, and availability. Consequently, resistance to the drug poses a threat not only to the treatment of OPC, but also to the management of systemic infections. In vitro fluconazole susceptibility has been shown to reliably predict the therapeutic outcome of OPC in both experimental models and clinical scenarios [29]. Standardised susceptibility testing for the *Candida* species-fluconazole combination closely approximates the predictive utility of antibacterial susceptibility testing which follows a “90–60 rule”: infections due to susceptible isolates respond to therapy 90% of the time, whereas infections due to resistant isolates respond 60% of the time [30]. The relationship between in vitro testing and therapeutic outcome is less clear cut in patients with invasive mycoses, such as candidaemia [31]. Odds et al. have likened susceptibility testing to weather forecasts which predict trends but “are unable to accurately foresee temperatures or levels of precipitation at a specific time in a specific location” [32]. Likewise, the complex interactions involving antifungal drug, fungus, and host in systemic disease means the susceptibility test result provides only a fragment of information necessary to predict the likely outcome of treatment. This piece of information is, nonetheless, vital; thus, we advocate routine speciation and sensitivity testing of clinically-relevant isolates as an adjunct to optimizing treatment [33].

This study is limited by a number of factors. The small number of isolates, with even fewer recovered from actual OPC cases, may have masked the magnitude of fluconazole resistance, especially in symptomatic patients. The single centre design limits the generalizability of the results which, thus, may not be widely applicable. It is also noteworthy that the method employed to differentiate *C. dublinensis* from *C. albicans* using colour on CHROMagar, as proposed by Messeir et al. [12], is subjective. Although this could have influenced the species distribution, it does not affect the susceptibility pattern which is the thrust of the study.

In summary, the prevalence of fluconazole resistance in our study is higher than rates obtained from similar studies previously conducted in other Nigerian cities. In view of this, and considering that OPC stems from organisms colonizing the oral cavity, we recommend surveillance of OPC lesions in HIV patients to better define the extent of fluconazole resistance. Future research should adopt a multi-centre approach to accommodate inter-regional variations.

## Figures and Tables

**Table 1:** In vitro fluconazole sensitivity profile of *Candida* isolates in PLWHA.

*Candida* Species	Sensitivity to Fluconazole Frequency (%)		
	Sensitive *n* = 53	S-DD ^1^ *n* = 4	Resistant *n* = 18
C. albicans	47 (90.4)	3 (75.0)	11 (61.1)
C. glabrata	1 (1.9)	0 (0.0)	3 (16.7)
C. krusei	0 (0.0)	0 (0.0)	2 (11.1)
C. parapsilosis	3 (5.7)	1 (25.0)	1 (5.6)
C. tropicalis	1 (1.9)	0 (0.0)	1 (5.6)
C. famata	1 (1.9)	0 (0.0)	0 (0.0)

^1^ S-DD = Susceptible Dose Dependent.

**Table 2:** Factors associated with fluconazole resistant isolates in PLWHA.

Variable	Resistant Isolate *n* = 18	Sensitive/S-DD ^1^ Isolate *n* = 57	χ²	*p*-Value
Past Fluconazole Use				
Present	16 (48.5)	17 (51.5)	19.368	<0.001
Absent	2 (4.8)	40 (95.2)		
History of OPC				
Present	13 (41.9)	18 (58.1)	9.319	0.002
Absent	5 (11.4)	39 (88.6)		
Species				
*C. albicans*	11 (18.0)	50 (82.0)	NA ^2^	0.032
NAC	7 (50.0)	7 (50.0)		

^1^ S-DD = susceptible dose-dependent; ^2^ NA = not applicable, Fisher’s Exact Test used; Percentages in parentheses represent row variable.

## Supplementary Materials

Supplementary File 1
